# Epidemiological Characteristics of *bla*_NDM-1_ in *Enterobacteriaceae* and the *Acinetobacter calcoaceticus-Acinetobacter baumannii* Complex in China from 2011 to 2012

**DOI:** 10.1371/journal.pone.0113852

**Published:** 2014-12-03

**Authors:** Weimei Ou, Lanqing Cui, Yun Li, Bo Zheng, Yuan Lv

**Affiliations:** The Institute of Clinical Pharmacology, Peking University First Hospital, Peking University, Beijing, 100191, P.R. China; Iowa State University, United States of America

## Abstract

**Objectives:**

The study aimed to investigate the prevalence and epidemiological characteristics of *bla*_NDM-1_ (encoding New Delhi metallo-β-lactamase 1) in *Enterobacteriaceae* and the *Acinetobacter calcoaceticus-Acinetobacter baumannii* complex (ABC) in China from July 2011 to June 2012.

**Methods:**

PCR was used to screen for the presence of *bla*_NDM-1_ in all organisms studied. For *bla*_NDM-1_-positive strains, 16S rRNA analysis and Analytical Profile Index (API) strips were used to identify the bacterial genus and species. The ABCs were reconfirmed by PCR detection of *bla*_OXA-51-like_. Antibiotic susceptibilities of the bacteria were assessed by determining minimum inhibitory concentration (MIC) of them using two-fold agar dilution test, as recommended by the Clinical and Laboratory Standards Institute (CLSI). Molecular typing was performed using pulsed-field gel electrophoresis (PFGE). S1 nuclease-pulsed-field gel electrophoresis (S1-PFGE) and Southern blot hybridization were conducted to ascertain the gene location of *bla*_NDM-1_. Conjugation experiments were conducted to determine the transmission of *bla*_NDM-1_-positive strains.

**Results:**

Among 2,170 *Enterobacteriaceae* and 600 ABCs, seven *Enterobacteriaceae* strains and two *A. calcoaceticus* isolates from five different cities carried the *bla*_NDM-1_ gene. The seven *Enterobacteriaceae* strains comprised four *Klebsiella pneumoniae*, one *Enterobacter cloacae*, one *Enterobacter aerogen* and one *Citrobacter freundii*. All seven were non-susceptible to imipenem, meropenem or ertapenem. Two *A. calcoaceticus* species were resistant to imipenem and meropenem. Three *K. pneumoniae* showed the same PFGE profiles. The *bla*_NDM-1_ genes of eight strains were localized on plasmids, while one was chromosomal.

**Conclusions:**

Compared with previous reports, the numbers and species containing the *bla*_NDM-1_ in *Enterobacteriaceae* have significantly increased in China. Most of them are able to disseminate the gene, which is cause for concern. Consecutive surveillance should be implemented and should also focus on the dissemination of *bla*_NDM-1_ among gram-negative clinical isolates.

## Introduction

Carbapenems are the mainstream treatment for antibiotic-resistant bacterial infections, especially infections triggered by multi-drug resistant gram-negative bacteria [1]. Therefore, the presence of carbapenemase, which can hydrolyze carbapenems, in clinical gram-negative organisms is an important threat to public health [2]. More worryingly, New Delhi metallo-β-lactamase 1 (NDM-1; encoded by *bla*_NDM-1_), a new type of carbapenemase, can hydrolyze almost all known β-lactam antimicrobials (except aztreonam), including the last resort carbapenems [3]. Bacteria carrying *bla*_NDM-1_ are thus referred to as “superbugs” by the media. This article focused on the problem of carbapenem resistance mediated by NDM-1.

NDM-1, a new type of Ambler class B metallo-β-lactamases (MBLs), was first reported in *K. pneumoniae* and *Escherichia coli* derived from a Swedish patient of Indian origin who was admitted to hospital in New Delhi, India in 2009 [4]. Since then, *bla*_NDM-1_-positive bacteria have been disseminated worldwide, including all seven continents, except Antarctica. The Indian subcontinent and China are the major reservoirs. Balkan states, such as Serbia, Montenegro and Bosnia-Herzegovina may be considered as a ‘secondary’ reservoir area, while the Middle East (Morocco, Algeria, Libya, Egypt, Iraq, Kuwait, Oman, Lebanon and Afghanistan), Southeast Asia (South Korea, Indonesia, Vietnam and Thailand) and parts of Europe (France, Italy) may be additional reservoir areas [5]. The *bla*_NDM-1_ gene has been detected in *K. pneumoniae*, *E. coli*, *Klebsiella oxytoca*, *Enterobacter cloacae*, *Enterobacter aerogenes*, *Proteus spp*., *Citrobacter freundii*, *Morganella morganii*, *Providencia spp*., *Acinetobacter spp*. *and Raoultella ornithinolytica* [6]–[24]. It is typically carried by plasmids, but can be chromosomal [25]. Plasmids carrying *bla*_NDM-1_ are mostly transferable and coexist with many other resistance determinants [12], [14], [18], further complicating the treatment of NDM-1-producing bacteria infections.

This study retrospectively surveyed the nationwide epidemiology of *bla*_NDM-1_ in *Enterobacteriaceae* and ABCs strains derived from the Ministry of Health National Antimicrobial Resistance Surveillance Net (Mohnarin) program in China, from July 1, 2011 to June 30, 2012. The Mohnarin program was established by the Ministry of Health in 2004 to obtain a more complete picture of bacterial resistance in China to provide scientific data for clinical antibiotic applications. Based on the surveillance hospital definition standards, including geographic distribution, clinical microbiological capability and hospital willingness, 18 hospitals located in 18 different cities around China were selected as the monitoring sites of Mohnarin. The Institute of Clinical Pharmacology, Peking University First Hospital, was assigned as the central laboratory for the Mohnarin organization and was, therefore, in charge of the surveillance, both coordinating the program and carrying out the minimal inhibitory concentration (MIC) analyses.

## Materials and Methods

### Bacterial strains

All strains in the study were collected from the Mohnarin program. The specimens were all passively collected from patients’ clinical samples in the program, in other words, the strains were collected from regular clinical examination without damaging the patients’ interest, and the patients’ personal privacies were protected. In total, 2170 *Enterobacteriaceae* and 600 ABCs were collected from 18 tertiary hospitals representing 18 different cities in China participating in the the Mohnarin program, from July 1, 2011 to June 30, 2012. The specimens were isolated from patients’ blood, urine, secretion, sputum, feces, drainage, throat swab, cerebrospinal fluid and others, mostly collected from intensive care units (ICU, 17.8%), non-intensive care units (NICU, 81.6%) (Some strains were not specified as being from ICU or NICU). The patients’ characteristics were male (61.6%), female (37.3%), the elderly (34.7%), adults (55.1%) and children (9.2%). Only the first strain of duplicated isolates from the same patient was collected. Further information about the patients, such as the underlying disease and possible antimicrobial pretreatment, were, unfortunately, not available. 338 *Enterobacteriaceae* and 395 ABCs that were not susceptible to carbepenem were selected from the 2170 *Enterobacteriaceae* and 600 ABCs in the program to detect the *bla*_NDM-1_ gene. Concurrent quality control strains used for antimicrobial susceptibility tests were *E. coli* ATCC25922, *E. coli* ATCC35218 and *Pseudomonas aeruginosa* ATCC27853. *Salmonella* serotype *Braenderup* strain H9812 was used as the marker for pulsed field gel electrophoresis (PFGE).

### PCR amplification

The DNA extraction was performed from fresh culture using the boiling technique. The primers used were based on primers published by the Chinese Center for Disease Control and Prevention (CDC), F: TCG CAT AAA ACG CCT CTG; R: GAA ACT GTC GCA CCT CAT [26]. The amplicons were sequenced and compared with the published sequence in GenBank database using multiple sequence alignment.

### Species identification

The bacterial genera of the *bla*_NDM-1_-positive organisms were identified by PCR amplification of 16S rRNA, using the universal primers 27F-AGAGTTTGATCCTGGCTCAG and 1492R-GGCTACCTTGTTACGACTT [27]. The PCR products were sequenced and compared with the published sequence in GenBank by multiple sequence alignment. To determine the species, a Kligler Iron Agar assay was carried out first to detect whether the bacteria were fermentative or not. The bacteria were further distinguished using Analytical Profile Index (API) 20E or 20NE tests (bioMe'rieux, Craponne, France) to further identify the bacterial species. The non-fermentative bacteria were identified using API 20NE, while the *Enterobacteriaceae* were identified using API 20E. The ABCs were distinguished by PCR detection of *bla*_OXA-51-like_, which is intrinsic to *A. baumannii*, using primers previously reported as F: TAA TGC TTT GAT CGG CCT TG; R: TGG ATT GCA CTT CAT CTT GG [28].

### Antimicrobial susceptibility

The MICs of the *bla*_NDM-1_-positive isolates and transconjugants were determined using the agar dilution method, according to the recommendations given in document M100-S23 of the Clinical and Laboratory Standards Institute (CLSI). The results were interpreted according to the CLSI2013 M100-S23 guidelines [29]. The breakpoints of imipenem and meropenem for *Enterobacteriaceae* are as follows: susceptible (S), ≤1 µg/ml; resistant (R), ≥4 µg/ml, ertapenem S: ≤0.5 µg/ml, R: ≥2 µg/ml, aztreonam S: ≤4 µg/ml; R:≥16 µg/ml. Likewise, the breakpoints of imipenem and meropenem for the ABCs are: S:≤4 µg/ml; R:≥16 µg/ml. Tigecycline was interpreted according to the USA-FDA breakpoint (S: ≤2 µg/ml, R: ≥8 µg/ml).

### PFGE

Bacterial DNA was prepared in agarose blocks and digested with restriction enzyme XbaI (four *K. pneumonia* species and *Salmonella* serotype *Braenderup* strainH9812) and ApaI (two *A. calcoaceticus* species*).* The DNA fragments were separated by use of a CHEF-Mapper XA PFGE system (Bio-Rad, Richmond, USA) at 6 V/cm and 14°, with a pulse angle of 120°, for 23 h and a switch time from 4 to 40 s for *Enterobacteriaceae* while 24 h and a switch time from 5 to 20 s for *A. calcoaceticus.* The gel was stained with ethidium bromide.

### S1-PFGE and Southern blot hybridization

According to literature [30], bacterial DNA was prepared in agarose blocks and digested with S1 nuclease, and then separated by PFGE with conditions of 14 h at 6 V/cm and 14°, with a pulse angle of 120° and a switch time from 1 to 10 s. The gel was stained with ethidium bromide. The DNA fragments were transferred to nylon membranes (Hybond N, Amersham, UK), hybridized with digoxigenin-labeled *bla*_NDM-1_-specific probes and detected using an NBT/BCIP color detection kit (Roche, Basel, Switzerland).

### Plasmid analysis and Southern blot hybridization

Plasmids were extracted by the SDS alkaline lysis method and then digested with EcoRI. The plasmid DNA fragments were separated by agarose gel electrophoresis at 90 v for 2 h. The gel was stained with ethidium bromide. The plasmid fragments were then transferred to a nylon membrane, hybridized with digoxigenin-labeled *bla*_NDM-1_-specific probes and detected using an NBT/BCIP color detection kit as above.

### Conjugation experiments

Liquid mating was performed for the seven (using only one strain of M194 from the same clone represented by M186, M187, M194) NDM-1-producing strains, using *E. coli* J53 (azide resistant) as the recipient strain, with selection on Mueller-Hinton Agar containing sodium azide and ceftazidime: sodium azide 200 µg/ml and ceftazidime 16 µg/ml for U091, Q297, X122, G113, X231; and sodium azide 300 µg/ml and ceftazidime 16 µg/ml for M194, Q442 according to pre-experiments. When liquid mating failed, filter mating was then conducted to further test their transmission. The donor and recipient strains were added at a proportion of 1∶10 for both liquid mating and filter mating. Transconjugants were confirmed by PCR, and by API 20E and antimicrobial susceptibility testing.

## Results

### The identification of *bla*_NDM-1_-positive bacteria

PCR identified nine *bla*_NDM-1_-positive strains. The sequencing results of the amplicons showed all were 100% identical to *K. pneumoniae* strain 05–506 (GenBank accession number: FN396876). Three of them were from Hunan Province, two from Shaanxi Province, two from Zhejiang Province, one from Xinjiang Province and one from Jiangsu Province (Table 1), located in the East and Northwest of China. 16S rRNA sequencing and biochemical API strips revealed that four were *K. pneumoniae* (M186, M187, M194, U091), two were ABCs (G113, X231), one was *Enterobacter cloacae* (Q297), one was *Enterobacter aerogenes* (Q442) and one was *Citrobacter freundii* (X122), respectively. *bla*_OXA-51-like_ detection in the two ABCs was negative, indicating that both G113 and X231 were *A. calcoaceticus.*

**Table 1:** Epidemiological characteristics of nine *bla*_NDM-1_-positive bacteria.

Strains	Year of isolation	Patient sex	Patient age	Specimen	Ward	Origin
M186	2012	male	adult	secretion	NICU	Hunan
M187	2012	male	adult	secretion	NICU	Hunan
M194	2012	male	adult	secretion	NICU	Hunan
U091	2011	male	adult	urine	ICU	Xinjiang
Q297	2011	male	adult	blood	NICU	Shaanxi
Q442	2012	male	elderly	sputum	NICU	Shaanxi
X122	2011	male	adult	urine	NICU	Zhejiang
G113	2012	female	adult	blood	ICU	Jiangsu
X231	2011	male	adult	urine	NICU	Zhejiang

### The MICs of *bla*_NDM-1_-positive bacteria

All nine strains were highly resistant to a broad spectrum of antibiotics, including piperacillin, Third- and Fourth-cephalosporins, β-lactamase inhibitor combinations, most carbapenems and nitrofurantoin; however, they showed varied susceptibilities to aminoglycosides and tetracyclines. Fortunately, most strains showed susceptibility to fluoroquinolones and tigecycline (Table 2).

**Table 2:** The MICs of *bla*_NDM-1_-positive bacteria.

Strains	PRL	CTX	CRO	CAZ	SCF	FEP	ATM	IMP	MEM	ETP	GEN	AMK	TCY	MNO	TGC	CIP	LVP	NIT	POL	POS
M186	>256	256	>256	>256	256	32	>256	4	8	32	0.5	1	0.5	1	0.5	0.031	0.031	128	0.25	2
M187	>256	256	>256	>256	>256	64	>256	4	8	16	0.5	1	1	1	0.5	0.031	0.031	128	0.5	4
M194	>256	256	>256	>256	>256	64	>256	8	8	16	0.5	1	2	1	0.5	0.031	0.062	128	0.5	4
U091	>256	256	256	>256	256	32	64	4	8	8	1	0.25	256	64	0.5	0.25	0.5	32	0.5	8
Q297	>256	>256	256	>256	>256	64	256	4	8	32	128	2	128	64	1	0.5	2	64	0.5	2
Q442	>256	>256	>256	>256	>256	64	256	2	4	16	0.25	1	256	128	4	4	4	128	0.5	4
X122	256	256	256	>256	256	32	64	2	2	8	32	0.5	128	16	0.5	8	8	16	0.5	0.25
G113	256	>256	>256	>256	128	>256	16	128	128	256	256	8	2	0.125	0.5	0.25	0.5	256	0.5	64
X231	256	>256	>256	>256	256	>256	32	128	128	>256	1	2	2	0.062	0.125	0.062	0.062	128	0.5	128
J53	1	0.062	0.031	0	0.125	0.031	0.062	0.125	0.016	≤0.004	0.5	1	2	2	0.25	0.004	0.016	8	1	0.5
J53-U091	>256	256	>256	>256	>256	64	64	2	4	4	4	1	32	2	0.25	0.125	0.25	8	2	0.5
J53-Q297	256	64	128	>256	64	8	64	8	4	8	0.25	1	2	2	0.25	0.004	0.016	8	1	0.5
J53-Q442	256	128	256	>256	128	32	32	4	4	8	0.5	1	2	2	0.25	0.008	0.016	16	0.5	0.5
J53-X122	256	128	256	>256	128	16	32	4	4	16	0.5	1	2	2	0.25	0.008	0.016	8	1	0.5
J53-G113	64	32	64	>256	32	2	0.062	1	0.5	0.5	0.125	1	2	2	0.25	0.004	0.016	8	1	0.5
J53-X231	32	64	32	>256	16	2	0.031	1	0.5	0.25	0.5	1	2	2	0.25	0.008	0.016	16	1	0.5

PRL: Piperacillin. CTX: Cefotaxime. CRO: Ceftriaxone. CAZ: Ceftazidime. SCF: Cefoperazone/sulbactam2:1. FEP: Cefepime. ATM: Aztreonam. IMP: Imipenem. MEM: Meropenem. PAN: panipenem. ETP: ertapenem. GEN: Gentamicin. AMK: Amikacin. TCY: tetracycline. MNO: Minocycline. TGC: Tigecycline. CIP: ciprofloxacin. LVP: Levofloxacin. NIT: Nitrofurantoin. POL: Polymyxin B. POS: phosphonomycin. USA-FDA breakpoint was applied for tigecycline (S: ≤2 mg⋅L^−1^; R: ≥8 mg⋅L^−1^) in both *Enterobacteriaceae* and *A. calcoaceticus*.

The transconjugants J53-U091, J53-Q297, J53-Q442 and J53-X122 were resistant to many antibiotics, including piperacillin, cephalosporins, β-lactamase inhibitor combinations and most tested carbapenems, but remained susceptible to tigecycline, ciprofloxacin, levofloxacin and polymyxin B. J53-G113 and J53-X231 did nor seem to be resistant to carbapenems; however, the MICs of the carbapenems were relatively high compared with those for the recipient *E. coli* J53 (Table 2).

### PFGE

**Figure 1:**
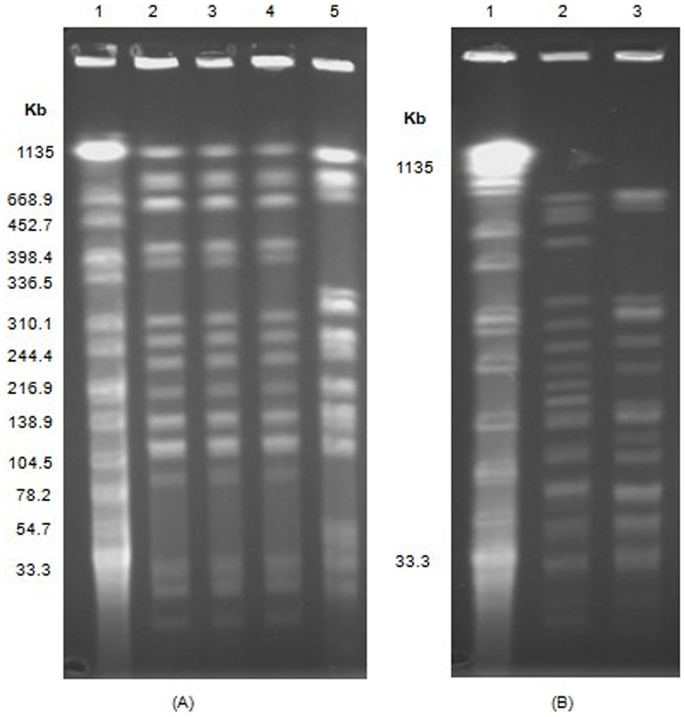
PFGE profiles of *Salmonella* serotype *Braenderup* strain (H9812), and four *K. pneumoniae* and two *A. calcoaceticus* isolates. (A) PFGE profiles of H9812 and four *K. pneumoniae* isolates. Lane 1: *Salmonella* serotype *Braenderup* strain (H9812), as the marker. Lane 2∼5: four *K. pneumoniae* isolates (M186, M187, M194, U091, respectively). (B) PFGE profiles of H9812 and two *A. calcoaceticus* isolates. Lane 1: *Salmonella* serotype *Braenderup* strain (H9812), as the marker. Lane 2∼3: two *A. calcoaceticus* isolates, G113 and X231, respectively.

Three *K. pneumoniae* from the same hospital (M186, M187, M194) had the same PFGE profiles, indicating they were the same clone, while *K. pneumoniae* U091 showed a different profile, suggesting U091 was another clone (Figure 1A). Two *A. calcoaceticus* (G113, X231) showed different profiles (Figure 1B).

### S1-PFGE and Southern blot hybridization

**Figure 2:**
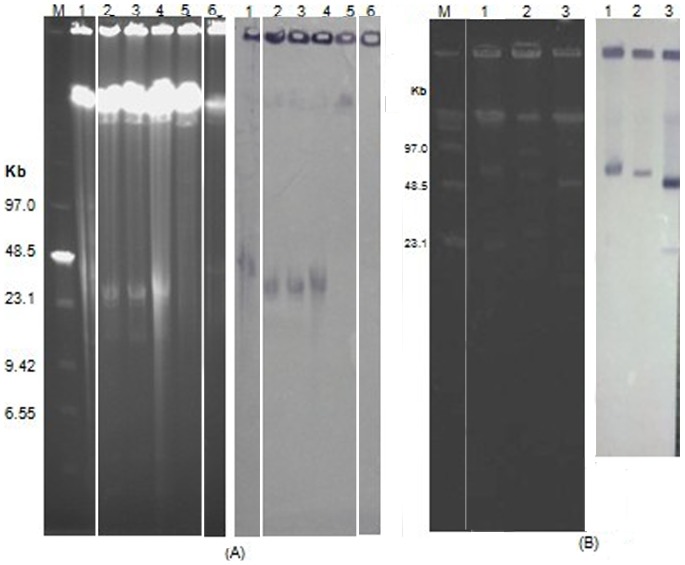
Result of S1-PFGE and Southern blot hybridization. (A) The left dark background figure shows the results of S1-PFGE, while the right light background shows the Southern blot hybridization. M: Low Range PFG Marker. Lane 1∼6: *A. calcoaceticus* G113, *K. pneumoniae* M186, *K. pneumoniae* M187, *K. pneumoniae* M194, *K. pneumoniae* U091 and *Citrobacter freundii* X122, respectively. (B) The left dark background figure shows the results of S1-PFGE, while the right light background shows the Southern blot hybridization. M: Low Range PFG Marker. Lane 1∼3: *Enterobacter cloacae* Q297, *Enterobacter aerogenes* Q442 and *A. calcoaceticus* X231, respectively.

From the hybridization results, we observed that seven strains (M186, M187, M194, Q297, Q442, G113 and X231) carried the *bla*_NDM-1_ gene on plasmids, whose sizes ranged from approx. 23 to approx. 80 kb. The *bla*_NDM-1_ gene in *K. pneumoniae* U091 was demonstrated as chromosomal. X122 failed to produce a *bla*_NDM-1_–specific fragment (Figure 2A, 2B).

### Plasmid analysis and Southern blot hybridization

**Figure 3:**
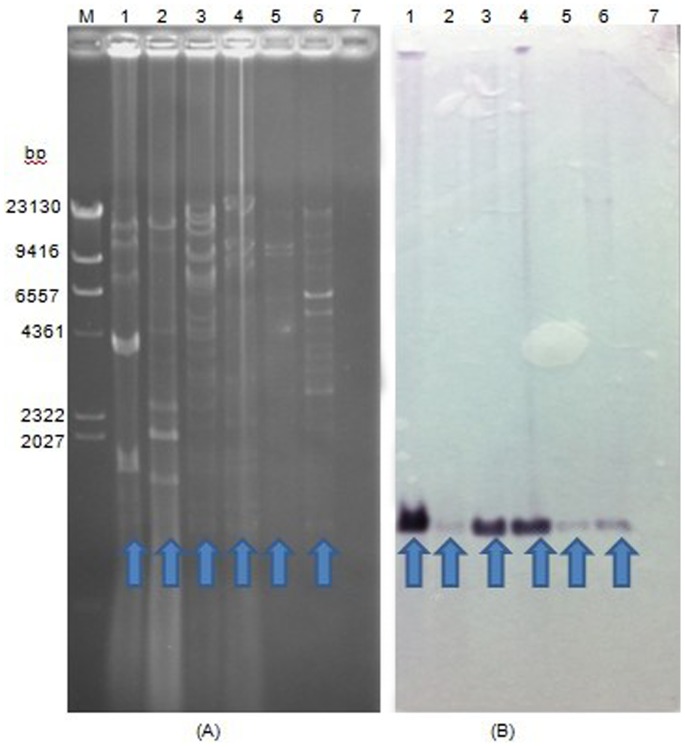
EcoRI-digestion profiles and Southern blotting results of extracted plasmids. (A) Plasmid digested with EcoRI and then run in gel. (B) Southern blot hybridization results of digested plasmid DNA. M: λDNA\HindIII. Lane 1∼7: *K. pneumoniae* M194, *Enterobacter cloacae* Q297, *Enterobacter aerogenes* Q442, *Citrobacter freundii* X122, *A. calcoaceticus* G113, *A. calcoaceticus* X231 and *K. pneumoniae* U091, respectively.

Plasmid extraction and Southern blotting was performed to further check the location of the *bla*_NDM-1_ gene in the studied strains. *K. pneumoniae* U091 did not have any reactive bands (Figure 3A), suggesting that U091 does not have any plasmids. This was consistent with chromosomal location of the *bla*_NDM-1_ gene inferred from the S1-PFGE and Southern blot results. The other six strains all had plasmids and the sizes of plasmids were consistent with S1-PFGE results shown in Fig. 2. All plasmid DNAs were digested with restriction enzyme EcoRI restriction enzyme, whose recognition site (5′ G∧AATTC 3′) does not occur in the sequence of the *bla*_NDM-1_ gene, as far as we know. Therefore, EcoRI should not cleave the *bla*_NDM-1_ gene. Figure 3B shows an approximately 2KB band in all positive isolates that corresponds to the *bla*_NDM-1_ gene plus flanking plasmid sequences. We concluded that the *bla*_NDM-1_ gene was located on the plasmids of the six strains that harbored plasmids.

### Conjugation experiments

Except for M194, the plasmids carrying *bla*_NDM-1_ from six strains were successfully transferred to *E. coli* J53. PCR confirmed the presence of the *bla*_NDM-1_ gene in the transconjugants and API 20E test confirmed them as *E. coli.* The MIC results are shown in Table 2. The plasmids of Q297, X122, G113 and X231 were transferred to *E. coli* J53 successfully by liquid mating, indicating that the *bla*_NDM-1_ gene from these strains can be disseminated easily, since liquid mating requires the strongest transfer ability among the three major conjugation methods: liquid mating, solid mating and filter mating. U091 and Q442 were conjugative by filter mating, while M194 failed.

## Discussion

In the past few decades, an alarming increase in the prevalence of antimicrobial resistant pathogens causing serious community- and hospital-acquired infections has occurred worldwide. The increase in carbapenem resistance in Gram-negative bacteria has become a major concern. Bacteria producing NDM-1 have caused a global panic because they can hydrolyze almost all known β-lactam antimicrobials (except aztreonam), including the last resort carbapenems [3], and are referred to as “superbugs” by the media. To further complicate matters, the *bla*_NDM-1_ gene (encoding NDM-1) has disseminated rapidly over distantly-related geographical areas around the world [11], [15], [19], [20]. In terms of human hosts, there are three major routes to acquire an NDM-1 producing organism: nosocomial, personal travel and community acquisition. The *bla*_NDM-1_-carrying bacteria have been reported as gut colonizers in humans with or without clinical symptoms. They can also survive in the local environment, which may result in humans acquiring the *bla*_NDM-1_-positive bacteria unconsciously [5]. Hence, bacteria possessing the *bla*_NDM-1_ gene constitute a serious human health threat.

The most common mechanism of resistance to carbapenem is the production of carbapenemases (one of β-lactamases), including enzymes of Ambler class A, D and B (MBLs). NDM-1 is an MBL that mediates carbapenem-resistance. In this study, we confirmed that none of the *bla*_NDM-1_-bearing strains were susceptible to carbapenem.

The Chinese CDC has reported *bla*_NDM-1_-producing bacteria since 2010. Since then, there have been many studies on this issue. Chen et al. reported four *A. baumannii* on mainland China [2], Ho et al. reported one *E. coli* in Hong Kong [18] and Wu et al. reported *K. pneumoniae* in Tai Wan [21]. To date, a number of *bla*_NDM-1_-positive bacteria have been reported in China, and the species has included *E. coli, K. pneumoniae, K. oxytoca, K. ozaenae, E. cloacae, E. aerogen, C. freundii, Salmonella enteritidis, Morganella morganii, Providencia spp., Alcaligenes faecalis, Kocuria varians, Moraxella group, Comamonas testosteroni, Stenotrophomonas maltophilia, Staphylococcus capitis, Methylobacterium species, Raoultella ornithinolytica, Acinetobacter spp.* and *E. faecium*. Although there have been several molecular level researches on the genetic context of *bla*_NDM-1_, there are limited recent studies on the epidemiology of *bla*_NDM-1_-containing isolates [1], [2], [9], [13], [16], [23]. Compared with previous reports [2], [13], the present study demonstrated that the numbers and species of *bla*_NDM-1_ in family *Enterobacteriaceae* have significantly increased in China, and include *Klebsiella pneumoniae*, *Enterobacter cloacae*, *Enterobacter aerogen* and *Citrobacter freundii.* This is worrying because *Enterobacteriaceae* are the main cause of nosocomial infections.

Our study reported nine *bla*_NDM-1_-producing strains, of which three were the same clone. Except the *K. pneumoniae* U091, whose *bla*_NDM-1_ gene is chromosomal, Southern blotting showed that the *bla*_NDM-1_ genes were carried on plasmid in the other strains, which may permit them to transfer the gene to other strains and species, resulting in rapid horizontal transmission. Thus, we conducted conjugation experiments to further determine their transmission. The transconjugants J53-U091, J53-Q297, J53-Q442 and J53-X122 were resistant to almost all β-lactams, even aztreonam (Table 2), which is stable to MBLs, including NDM-1. This may be explained if more than one plasmid was transferred into *E. coli* J53 or the transferred element contained not only *bla*_NDM-1_ gene, but also other resistance determinants. Q297, X122, G113 and X231 may disseminate easily, because they were conjugative by liquid mating, which requires the strongest transfer ability among three major conjugation methods: liquid mating, solid mating and filter mating. This result should attract much attention. Interestingly, S1-PFGE, Southern blotting and plasmid extraction showed that U091 has no plasmids, yet the conjugation experiment was successful. It may be that its *bla*_NDM-1_ gene is carried on a transposon in the chromosome, which requires further study. Sequencing of the *bla*_NDM-1_ harboring plasmids or the flanking regions of the gene is required to better understand the genetic environment and transmission of this important multidrug resistance gene.
